# Evaluation of through-time radial GRAPPA for functional cardiac imaging in a patient population

**DOI:** 10.1186/1532-429X-16-S1-W12

**Published:** 2014-01-16

**Authors:** Nicole Seiberlich, Gunhild Aandal, Victoria Yeh, Trevor Jenkins, Mark A Griswold, Robert Gilkeson, Vikas Gulani, Prabhakar Rajiah

**Affiliations:** 1 Biomedical Engineering, Case Western Reserve University, Cleveland, Ohio, USA; 2 Haraldsplass Deaconess Hospital, Bergen, Norway; 3 Case Western Reserve University School of Medicine, Cleveland, Ohio, USA; 4 Cardiovascular Medicine, Harrington Heart & Vascular Institute, University Hospitals of Cleveland, Cleveland, Ohio, USA; 5 Radiology, University Hospitals of Cleveland, Cleveland, Ohio, USA

## Background

Real-time cardiac imaging with through-time radial GRAPPA has been shown to yield high quality functional images [Seiberlich, et al. MRM2011 Feb;65(2):492-505]. The goal of this work is to evaluate through-time radial GRAPPA in terms of overall scan time, quantitative measures of ESV, EDV, and EF, and image quality in a patient population.

## Methods

63 patients scheduled for routine CMR examinations were scanned on a 1.5T Siemens Avanto scanner with 12-18 receiver coils in this IRB and HIPPA-compliant study. The gold-standard gated and breathheld short-axis cardiac functional examination was performed for each patient with a temporal resolution between 31 and 62 ms, GRAPPA R = 2, and an in-plane resolution between 1.4-2.6 mm^2^. Real-time, ungated and free-breathing scans with through-time radial GRAPPA were also performed with a radial bSSFP sequence, temporal resolution of 42.2 ms, in-plane resolution of 2.3 mm^2^, and a reduction factor of 8 (16 projections for a 128^2 ^matrix). Either 26 (23 patients) or 6 (40 patients) calibration frames were collected for the through-time radial GRAPPA scan. The total scan times were noted for each patient and type of scan. ESV, EDV, and EF values were computed for both the standard and the real-time cardiac datasets and evaluated for agreement using two-sample t-tests. Images were rated for specific features on a scale of 1 (no visibility) to 4 (excellent) by two board-certified cardiac imagers. The distributions of review ratings were compared using the Wilcoxon test. A two-sample t-test was performed to compare the scan durations.

## Results

**Figure 1:**
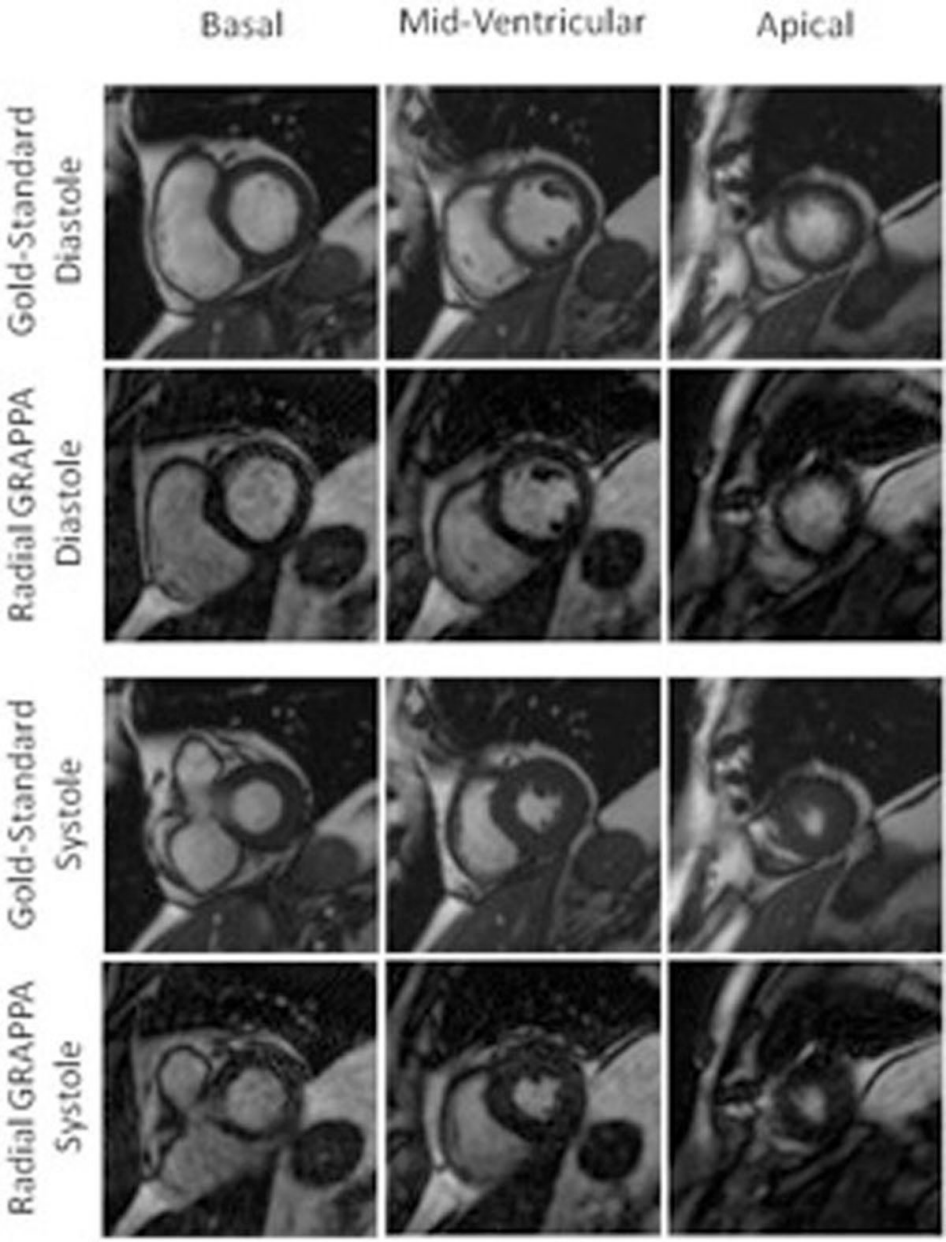
**Example images generated using standard functional imaging (first, third rows) and free-breathing, ungated through-time radial GRAPPA with 6 calibration frames (second, fourth rows) in both diastole (top) and systole (bottom) for three different slice positions**.

Examples images are shown in Figure 1. The results of the statistical analyses are shown in Table 1. The measures of ESV, EDV, and EF based on the breathhold gold-standard images and the real-time radial GRAPPA images are statistically equivalent. Similarly, statistical analysis showed that the image quality scores for the endocardial border definition, myocardium, cardiac motion were equivalent for the two methods. The raters weakly preferred the standard images for visualizing papillary muscle when only 6 calibration frames were used, and strongly preferred the standard images for blood pool and mitral valve visualization in all cases. Scan time was significantly shorter when using through-time radial GRAPPA with either 6 or 26 calibration frames, where the averages were 5.7 min with gold-standard imaging vs. 4.7 min with radial GRAPPA using 26 calibration frames and 2.8 min with 6 calibration frames.

**Table 1:** Results of statistical analyses for different image features

		Gold-Standard vs. Radial GRAPPA	Gold-Standard vs. Radial GRAPPA with 6 Cal Frames	Gold-Standard vs. Radial GRAPPA with 26 Cal Frames
Quantitative Parameters	EF	0.77	0.90	0.73
	
	ESV	0.97	0.93	0.87
	
	EDV	0.82	0.78	0.99

Qualitative Parameters	Endocardial	0.23	0.05	0.73
	
	Papillary	0.03 *	0.02 *	0.71
	
	Blood Pool	< 0.001 *	< 0.001 *	< 0.01 *
	
	Mitral Valve	< 0.001 *	< 0.001 *	< 0.001 *
	
	Myocardium	0.45	0.07	0.17
	
	Cardiac Motion	1.00	0.23	0.14

	Scan Duration	< 0.001 *	< 0.001 *	< 0.001 *

* denotes statistically significant differences

## Conclusions

Real-time, free-breathing functional cardiac imaging using through-time radial GRAPPA offers EF values equivalent to gold-standard cine imaging with shorter overall scan times in a general patient population. While the use of more calibration frames for through-time radial GRAPPA slightly improves the image quality, as few as 6 calibration frames per slice can be used to reduce functional imaging time to less than 3 min.

## Funding

NIH (R00EB011527, RO1HL094557, and UL1 RR024989) and Siemens Medical Solutions.

